# Preface

**DOI:** 10.1054/bjoc.2001.1754

**Published:** 2001-05


British Journal of Cancer (2001) **84**(Supplement 2), 17
© 2001 FNCLCC
doi: 10.1054/ bjoc.2001.1754, available online at http://www.idealibrary.com on IDEAL®

### Preface

The SOR project has developed a comprehensive series of clinical practice guidelines (CPGs) relating to the initial management of over 45 tumour types and clinical situations in cancer patients. All SOR documents follow the same format. Each clinical question is considered in sections corresponding to diagnosis, staging, treatment and follow-up. They comprise a detailed review and critical appraisal of published data with comparative tables and define the 'Standards', 'Options' and 'Recommendations' for each subject (see Figure 3, Project methodology). The full version of the SOR documents are important databases, comprising 50–300 pages depending on the subject, with up to 250 references and, when they exist, corresponding Medline abstracts.

The references for the full version of all SOR documents are listed in Appendix I.

At the end of each SOR document, the key standards, options and recommendations are summarized to provide a convenient practical guide for the management of each cancer type.

This supplement presents a selection of these summarises with treatment algorithms for those tumour types in which the guidelines have undergone external review and recent updating. All guidelines statements are 'Recommendations' according to the SOR definition (Table 1 Project Methodology) unless specified as 'Standards' or 'Options'. When Standards, Options or Recommendations are based on grade A, B or C evidence, this is clearly stated (see Table 2 Project Methodology). All other statements are based on lower quality evidence or expert agreement.

The SORs are integrated into clinical pathways that are presented in the form of algorithms or decision trees (Figure 1). They provide a graphic representation of a situation, focusing on specific clinical decisions that must be made at each stage in the management of a particular disease, i.e. in prevention, diagnosis, staging, treatment and follow up.

The algorithms are designed to help resolve clinical problems and to aid the clinician in choosing the most appropriate initial and subsequent plan of action. An algorithm always starts with a problem (a symptom or a specific clinical situation) and leads the reader through a series of dichotomous choices based on factors such as staging, prognostic indicators, medical history and clinical signs. It also describes the standards and options of subsequent actions (e.g. investigations or treatments) resulting from these choices. The basic schema for each algorithm involve boxes and arrows. The boxes contain phrases describing clinical conditions, criteria for choices, the decisions to be made and medical actions. They are of different shapes depending on whether they represent clinical conditions, lead to a decision (or evaluation) or describe the standards and options of the action to be taken.

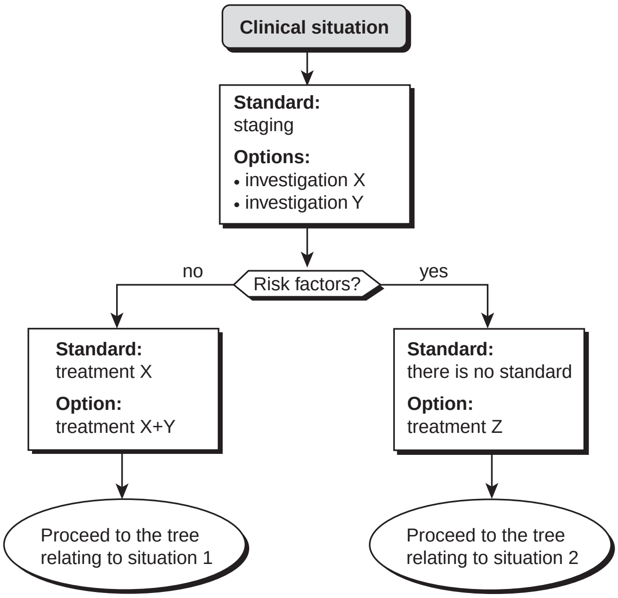

**Figure 1** Treatment algorithm structure. **Shaded rectangle with rounded corners**. These are found at the beginning of an algorithm to state the initial problem or clinical situation and in the middle of an algorithm when it is necessary to specify a certain clinical situation that arises from clinical tests or treatment. **Hexagons**. The decision criteria such as operability or risk factors. In general these lead to a binary choice with yes or no answers. **Rectangles**. These contain the standards and options relating to investigations, staging tests and treatments, etc. **Ovals**. These refer onto the next appropriate algorithm.

